# In between the lines of the narrative map: Phenomenological analysis of war rape victims in Amhara Regional State, Ethiopia

**DOI:** 10.1371/journal.pone.0289106

**Published:** 2023-07-28

**Authors:** Yemataw Wondie, Waganesh A. Zeleke, Mekides Melesse

**Affiliations:** 1 Department of Psychology, University of Gondar, Gondar, Ethiopia; 2 Department of Rehabilitation Counseling, Virginia Commonwealth University, Richmond, Virginia, United States of America; UBC: The University of British Columbia, CANADA

## Abstract

Research conducted in the last four decades on rape and other forms of sexual violence shows that they are not only the result of transgression behaviors of some people but have been used as strategic, systemic, and calculated tools of war, ethnic cleansing, and genocide. Examining the nature and effect of wartime rape and sexual violence based on their distinctive features, context, and historical background is essential for research and service providers. This paper explores the lived experiences of sexual violence and gang rape victims during the North Ethiopian war in Amhara Region, Ethiopia. Data were collected from three girls and women survivors using a trauma and socio-culturally informed phenomenological approach. The finding shows that participants experienced a broad and complex range of psychological, physiological, emotional, and relational suffering after the rape. The result also shed light on some risk factors such as lack of awareness of the effect of traumatic events, stigma related to rape, and lacks victim protective legislation risk their journey to healing. The paper further discussed individual and community mental health responses for victims of war rape in culturally responsive and resource-poor settings.

## Introduction

There is no consensus definition of wartime sexual violence. The terms rape, sexual assault, and sexual violence are frequently used interchangeably. Rape refers to the perpetrator invading a person’s body by conduct resulting in penetration, however slight, of any part of the body with a sexual organ or of the anal or genital opening of the victim with any object. The invasion should be committed against a person incapable of giving genuine consent [1].

Given the nature of wartime rape and sexual violence, surrounded by insecurity and taboo, it is not easy to establish the degree of such torture. The prevalence rate of wartime sexual violence has been documented in various countries. This includes 39% of women during the Rwandan genocide, 25% of women in Azerbaijan, 19% of women in Burundi, and 11% displaced women in Cambodia [2, 3]. The rate of wartime sexual violence among women and girls has also been reported to be 33.5% in Liberia and 9% in Sierra Leone [4]. Two studies estimated the prevalence of wartime sexual violence in the eastern Democratic Republic of Congo to be 40% in women and 24% in men [5].

In conflict settings, rape and sexual violence are used as strategic, systematic, and calculated tools of war, ethnic cleansing, and genocide. They are used to intimidate women and communities, destroy specific groups’ social fabric and cohesion, and even as a final act of humiliation before killing the victim [6]. Hence, such crimes are different from considering rape and sexual violence as a byproduct of war or transgression by a few bad people. Rape and sexual violence have been documented in various combat and conflict settings worldwide and are considered modern genocide. This includes but is not limited to World War II, where both the allied and the axis forces committed rape; as the instrument of ethnic cleansing in the former Yugoslavia and as a means of genocide in Rwanda. In such contexts, belligerent parties have been using rape as a tool to punish, terrorize, and destroy communities. For instance, the International Criminal Tribunal of Rwanda prosecuted Jean-Paul Akayesu on charges of raping women and girls during the Rwandan genocide [7].

Wartime rape and sexual violence take different forms. These include rape, sexual slavery, forced prostitution, forced pregnancy, forced sterilization/abortion, sexual mutilation, and sexual torture [7]. In its severest form, wartime rape takes gang rape, where a woman or a girl is raped by several perpetrators, and public rape, where a woman or a girl endures an individual or gang rape in front of other people such as family members or relatives. When rape is committed in public, the impact is multifaceted ranging from the victim to those forced to see such an immoral act. Therefore, wartime rape and sexual violence are different from other forms of sexual violence. They are used as a weapon to terrorize and induce fear and control the civilian population. Such violence is viewed as torture, a war crime, and a crime against humanity that has prolonged psychological and social effects on individuals and society; it breaks up families and destroys the community. It is customary to see surface-level prevalence and incidents of wartime rape and sexual violence reported by social media, mainstream media, and humanitarian organizations. Research on media representation of women’s experience in a conflict zone (Iraq, Syria, and South Sudan) shows that women are represented as casualties of sexual violence, injury, or death than reflected in the full-lived experience of the victims [8, 9]. However, the deeper psychological, emotional, spiritual, and cultural values and costs lost for such crimes usually remain the missing core components.

Wartime rape and sexual violence bring various acute and long-term effects. A study from northern Uganda revealed that women survivors continue to suffer from unresolved and untreated trauma, lack access to mental health care, and face economic hardships due to community stigma and customary laws that prevent women from owning land [10]. The study also reported that such impacts were not limited only to the survivor women and girls but also affected their family members who witnessed the wartime rape and sexual violence. Such effects include secondary traumatic stress, memory repression, faith, and disrupted relationship after survivors disclosed their experiences or if they had children born of wartime rape [10]. A systematic review on war-related sexual violence involved 20 studies drawn from six countries, five in Africa, from which 18 studies were drawn. The study reported physical outcomes (i.e., pregnancy, traumatic genital injuries/tears, rectal and vaginal fistulae, sexual problems/dysfunction, and sexually transmitted diseases), social and mental health outcomes (i.e., post-traumatic stress disorder, anxiety, and depression), social outcomes such as rejection by family and/or community and spousal abandonment [11].

### The current study

The recent Ethiopian civil war caused devastating human and infrastructural consequences to millions of Ethiopians. Following a unilateral ceasefire and withdrawal of the national army from the Tigray region on June 28, 2021, the Tigray forces took control of parts of the neighboring Amhara and Afar regions for over five months. During these times, the Tigray forces made tremendous human and infrastructure destructions in these part of the country, such as summary killings, kidnapping, rape, gang rape, sexual assault of women and girls, and other severe sexual acts of violence that are considered war crimes and crimes made against humanity [12].

In any traumatic situation like this civil war, the traumatic event, the traumatic experience, and the traumatic effect should be considered. However, stories about wartime outbreaks of violence are designed to fit the media and only show what happened, separating the event from the individual or community experience. This may be because such events are surrounded by insecurity and taboo in wartime rape and sexual violence, especially in traditional, conservative, and religious societies like Ethiopia. The current study aims to narrow the gap in the theory and literature by exploring the untold story and the story between the lines of the victims of sexual violence and gang rape in the recent North Ethiopia war using cultural and contextual frameworks.

Hence, we used a phenomenological qualitative research approach that aims at the essence of the experience of women and girls who survived rape and sexual violence and the context in which they experience these problems. This study is, therefore, designed to answer the following research questions.

1. What is the lived experience (lived body, lived time, lived space, and lived others) of wartime rape victims in Amhara Reginal State?
2. What risk factors exist in girls’ and women’s environment (s) victimized by wartime rape and sexual violence?
3. What protective factors appear or need to be constructed in the environment (s) of girls and women victims of war rape and sexual violence?

## Material and methods

This study is guided by the phenomenological approach using the Vancouver School of doing Phenomenology (VSP), a unique combination of phenomenology [13], hermeneutic/interpretive phenomenology [14], and constructivism [15]. VSP, as a philosophical and methodological approach, focuses on the production of reconstructed understandings and a commitment to studying the world from the interacting individual point of view. As a research method, it involves the hermeneutic circle of grasping the meaning of the phenomenon by understanding the part and the whole. In this method, emphasis is placed upon seeing all individuals in their context and understanding that each person uniquely perceives the world. The research process in the VSP involves seven stages; the cyclic process of silence, reflection, identification, selection, interpretation, construction, and verification [16].

### Methodological context of the current study

A team of mental health professionals involving eight members from the University of Gondar was formed to provide crisis intervention to the internally displaced persons from different villages of Chena, north Gondar zone. Chena district is found near Debark town and is one of the hotspots of the war where the Tigray rebel forces committed grave atrocities such as mass and summary killings, rapes, gang rapes, and related sexual violence on innocent civilians [12]. Before the team traveled to the site, one-day training on psychological first aid, psychosocial support, ethics, and social networking was organized and given to the members. After the training, the team was deployed to respond to the community’s immediate psychological and medical needs in Chena.

This crisis intervention team from the University of Gondar made a door-to-door psychological assessment of 70 households in Chena Kebele, where the TPLF forces committed mass killings, rape, gang rape, and massive infrastructural distractions. The goal of the screening and assessment was to identify family members requiring immediate psychological and medical attention. Accordingly, the team identified three groups based on severity of the primary psychological and medical needs. These groups include individuals who experienced sexual violence or gang rape, those who lost their loved ones, and individuals who experienced physical violence and witnessed traumatic events. The team provided crisis intervention that included psychological first aid, brief counseling, and community-based psychoeducational group intervention for five days to each group.

There was a female social worker assigned at China Keble who first identified the victims. On a Sunday morning, the social worker announced that there was a psychosocial support team from the University of Gondar to provide service to those who were raped, gang raped, and sexually abused by the Tigray rebel forces. This set the stage for the psychosocial support team to give the support services where the lead psychology faculty were able to identify the three victim participants who gave consent for further interview.

### Sample and sampling procedure

Using purposive sampling, three women and girls from Chena, north Gondar zone, were selected based on the crisis intervention team leader’s judgment and the purpose of this research [17], and data was gathered from these participants from December 08 to 18, 2021. These interviewees are the primary unit of analysis. The reason behind recruiting the participants was to make the voices of all the victims in the region heard so that appropriate interventions would be provided and further research would be conducted. The participants were interviewed, and all the data was gathered in two different sessions within the first month after the sexual violence happened. While trust building and rapport were established during the first session by requesting some demographic information, more emotionally profound information was gathered during the second session. Since the informants were uncomfortable with any type of recording due to social stigma, no device was used during the interview.

### Instrument

#### In-depth interview

A self-developed semi-structured in-depth phenomenological interview was used to collect data from the participants. The questions were directed at the participants’ lived experiences, feelings, beliefs, and meaning about the phenomena and the setting. It addresses how the participants think and feel most directly by focusing on what goes on within the participants’ bodies, minds, time, and relationships. The interview questions include: What happened to you when the TPLF forces captured Chena? What are the feelings and thoughts you experienced after the sexual violence? Did you share your experience with anyone (family, friends, etc.)? How has the experience affected your body and relations with others etc.?

Follow-up questions were used to capture detailed stories. This allowed us to unfold the meaning of the participants’ experiences. As Creswell [17] described, the phenomenology study intends to understand the phenomena in the subject terms and explain the human experiences as the person herself experiences them.

#### Memoing

Given the participants’ position to participate in the study without being audio/video recorded, we used field notes recording what the interviewers heard, saw, experienced, and thought while collecting and reflecting on the process using narrative exposure therapy. We gave this therapy while interviewing the victims by which we had prepared a rope, a flower and a stone where the rope represented a life line, the flowers represented memories that are considered good or situations that were supportive to the participant while the stone represented traumas and adverse experiences of the participant. After the stones and the flowers are assigned and labeled by the participants, they were asked detailed questions about each experience. The participants gave their consent that their answers could be recorded by a notebook. In each experience the participants were asked to talk about details of their feelings and sensations in each adverse experience. It should also be noted that all the interviews were carried out in Amharic, the local and the official national language.

## Data collection procedure

This study used a phenomenological approach to examine interviews to understand the lived experience of war rape victims. The goal of the phenomenological method is to explain things from the perspective of those who actually experience them [17]. Among different phenomenological frameworks, we followed the 12 steps of the VSP research process to collect and analyze the data (see Table 1) with some modifications due to the cultural, situational, and language factors of the research process applies. Culturally, girls and women in the rural Amhara region are accustomed to brief dialogues or conversations with authority figures. Because of this, the crisis intervention team leader who traveled from the city conducted only two interviews with each participant. Furthermore, the data collected during the first month after the sexual violence happened in Amharic and the data coding and analysis made in English, we were unable to go back to the participants and verify the essential structure of our results. The construction of the central theme from the data was framed using Van Manen’s [18] existential analytical categories such as lived body, lived time, lived space, and lived others and biological analysis of risk and protective factors [19].

**Table 1:** The application of the 12 steps of the Vancouver School of doing phenomenology.

*The VSP 12 steps of the Research Process*	*Steps were taken in the current Study*
1. *Selecting dialogue partners (the sample)*	Three participants were selected through purposive sampling.
2. *Silence (before entering a dialogue)*	The researchers’ preconceived ideas and biases were deliberately put aside.
3. *Participating in a dialogue (Data collection)*	The research team conducted two in-depth interviews with three participants.
4. *Sharpened awareness of words (data analysis)*	We made the data analysis obtained from the three victims.
5. *Beginning consideration of essences (coding)*	The two researchers repeatedly try to answer the question, “what are the essences each participant says?”
6. *Deconstruction of the text and constructing the essential structure of the phenomena from the cases (individual case construction)*	The main factors of each case were highlighted using van Mannen’s lived existential framework, and the foremost essential factors were used to develop each case’s construction.
7. *Verifying each case construction with the relevant participant (verification)*	This was carried out with each participant interviewed individually
8. *Constructing the essential structure of the phenomenon from all interviews (meta-synthesis of the interviews)*	Two researchers participated in the data analysis and developed the common themes that emerged across all participants using cross-case analysis.
9. *Comparing the essential structure of the phenomena with the data (verification)*	Multiple readings across each case interview were performed.
10. *Identifying the overriding themes that describe the phenomenon (construction of the central theme)*	The category that emerges from the story of the victims was made using van Manen’s [16] existential analytical category and bioecological analysis of risk and protective factors.
11. *Verifying the essential structure with the participants (verification)*	Due to language and time factors, the researchers could not verify the essential structure with participants.
12. *Writing up the finding (reconstruction)*	Write up of the finding was made by two of the researchers.

### Ethically and culturally relevant strategies

Permission and ethical approval to conduct the research was obtained from officials at the University of Gondar, and the Ethical Committee of the College of Social Sciences and Humanities where the psychosocial support group was drawn. While a letter to provide psychosocial support was secured from the University officials, ethical approval was obtained from the Ethics committee of the college. We made use of informed consent cautions that deception may be counterproductive. However, not asking the central research questions is not regarded as deception. The ‘informed consent agreement’ was explained orally to participants at the beginning of each interview. We ensured that the participants understood the purpose of the study and how their responses would be used. They were informed that they can withdraw from the interview process and decline to respond to any questions they do not feel like responding to. In addition, the following considerations were taken care of to adhere to the ethical and cultural fitness of the study. The interviews were conducted intentionally by a woman. A constant check-up on the participants’ emotional state during the interview and a debrief after the interview was done. The participants received any necessary counseling from trained counseling and clinical psychologists from the University of Gondar. Interviews were translated into English by the researchers.

#### Trustworthiness

The following steps were taken to make sure the study’s credibility, transferability, dependability, and reflexivity. The interview guide was initially created by examining a substantial body of literature prior to performing the interview. Sensitive to cultural differences of the interview guide was checked. The interviews were done in Amharic, the mother tongue of the participants, by the lead psychosocial support team. The findings were totally based on participant descriptions rather than any researcher’s biases. This is backed by the final report, which further supports the study’s conformability and reflexivity by anecdotes of the participants. Additionally, the extensive data analysis procedure that demonstrates how the van Manen phenomenological analysis was completed using similarities and differences of the themes supports the internal coherence and validity of the themes and upholds the credibility of the study.

## Findings

Data about the lived experience of three participants who survived the wartime rape, gang rape, and sexual violence in Chena, North Gondar Zone, and the Amhara region was collected using in-depth interviews. To maintain the anonymity of the interviewees, their names are changed, and the details are withheld. The two girls, ’Emnet’ and ‘Tesfa’- were aged 15 and 19, respectively, at the interview. ‘Rebka,’ aged 56, was married and had seven children before the war. She did not know about her husband, who was deployed to the war as a militia group to fight the TPLF rebel forces. While Emnet was a 7^th^ grader before the war and lost her father during the war, Tesfa was a 10th grader before the war. These girls and women identified themselves as ethnic Amhara and Orthodox Christians. This study draws empirical findings from interviews with girls and women in the Amhara region (see Table 2).

**Table 2:** Socio-demographic background of the participant.

Case #	Age	Gender	Educational status	Ethnicity	Residency
Emnet	15	F	7^th^ grade	Amhara	North Gondar
Rebeka	56	F	No formal education	Amhara	North Gondar
Tesfa	19	F	10^th^ grade	Amhara	North Gondar

### Participants interview analysis

The following section presents selected key vital text from the participant’s answers to the in-depth semi-structured interview share. This information is presented in a way explored in this study to reflect each individual’s lived experiences regarding war-based sexual violence.

#### Participant 1

Ement, the 15 years old girl, was raped in her own home by several TPLF soldiers. She described the movement as, “…*at first*, *the TPLF soldiers arrived at our home unexpectedly*. *We cooked for them for some days*. *However*, *we wanted to escape*. *Thus*, *my mom and I tried to escape from them*. *They caught us and took me away*. *They told my mom to go alone*. *They told me that I would be staying with them or they would kill me*. *I did not get a chance to refuse since they were abusing me physically*. *They took me home*. *There I found some of my friends*. *They were also captives*.*”*

Ement reported that she was raped by one of the soldiers and witnessed other girls being rapped by other soldiers in the same situation. The rape happened during the night, followed by severe verbal abuse and cursing. “*In the middle of the night*, *they came to us and took one girl at a time*. *That night*, *the guy that caught me raped me*.*”* Emnet escaped the place the following day alone since other girls were scared to death to join her in her decision. She said: “*I told my friends that I plan to run away at any cost*. *They were scared*, *and they told me I might get killed*, *and they were afraid to do so*. *Then I waited until the Juntas were busy with other tasks*. *I was able to escape*. *As there was a jungle everywhere in Chena*, *I was able to run without being seen*. *It took me 5 hours to run and roll down from the mountains*. *I never felt any pain until I arrived at Dabat after 5 hours of running*. *I went to my aunt*. *Then I sent a message to mom that I was able to escape and that I was okay*.*”*

Emnet finally managed to get back home and met her mother when the area got free from the Tigray invaders. This was not, however, a less harrowing experience for her. She said, *“After Chena was free from the TPLF force*, *I went home*. *However*, *the house reminded me of all the abuse scenes*.*”* The support she received from her mother was very much instrumental. It was beyond her imagination to experience all those sufferings in her life.

As the saying goes, "adding salt on a wound," Emnet did not believe her father was killed in the war. She stated the experience and all the associated feelings: *“What was worse was that I found out that my dad was killed later*. *He went to fight with the TPLF force to protect his family and his community*. *However*, *he was not able to protect us*. *He died*. *I love my dad a lot*. *All I wanted was to study hard to make my dad proud of me*. *I was so sad because of the death of my dad*. *It still irritates me*. *I was not able to sleep for a month*. *Currently*, *I experience difficulty falling asleep if I recall my dad*. *I recall my dad calling my name with care and love*. *He always showed me love*. *He was never angry at me*. *I always recall him whenever I go to Chena*. *And it is hard for me to accept that he is gone and will never return*. *It irritates me that my family experienced double damage*.*”*

In Emmet’s view, what was more painful was not experiencing the sexual violence per se but the social stigma she faced afterward. The stigma ranges from child shepherds to adult community members who knew about the fact that the Tigray rebel forces raped her. Such stigma forced her to move from her village to another area where no one knew her condition. She said: “*In addition to the sexual abuse*, *the community started laughing at me*. *Whenever I pass by our neighborhood*, *they will loudly call me* የጁንታ ትራፊ *“leftover of the junta*,*”I hated myself because of my experience of abuse*. *I hated the community*. *I was stressed and irritated*.*”*

In all these ups and downs, Emnet gives credit to her mother. She said, *“My mom was my strength*. *She told me that I could have been more hurt if they had done the same thing to her*. *And she told me that I sacrificed myself to save her*.*”* Unfortunately, Emnet was forced to leave her mother to escape the community stigma. She stated: “*I love being around her*, *but the shepherds and the community were harsh*. *Thus*, *I was not able to resist*. *Because of that*, *I moved to a different community called Wokin*. *It is not easy to live in Wokin*. *Yet I felt better since I was far from the people that were insulting and mocking me*. *I do not want to see the village now*. *It has too many bad memories for me*.”

Finally, Emnet got relieved after she had the opportunity to do a medical check-up. She was happy for a couple of reasons. One is that she did not conceive, and the other is that she met several other women victims of rape and got a sense of *“*I was not alone.” She said:

*“The first time that I felt safe was when I was able to have a medical check-up at Debark hospital* and *when they told me that I was okay*. *I felt okay after meeting with women with similar experiences*. *I understood it was not only me*, *and I was not the target*, *but rather the community was a target*.*”*

#### Participant 2

Rebeka is a 56-year-old woman and a mother of seven children. She stated that she was home with her five children when the Tigray rebel forces arrived at Chena. Her husband was on the war front fighting the TPLF forces. She describes the moment and the brutal sexual violence that happened to her by seven TPLF soldiers “They *broke into my home*. *They all raped me until I was not able to move my body*. *They did anal sex*, *also*. *My children were watching all the scenes*. *I was not able to be fully conscious until the next day*.*”* Rebeka developed a serious physical health problem due to this horrific experience. For her, the most painful part was the psychological wound that came from the crime and the inhuman reaction to her. She said, “After that time, I cannot control *my urine and stool/ordure*. *It is not the rape that hurt me nor their ethnic slurs and beating; my children watched all this*, *and I cannot work and feed them now*. *I am old enough to be a grandmother*, *but I could not protect my kids*. *At my age*, *I do not know how to express what I feel about being a victim of seven men and developing Fistula on both sides*.”

For Rebeka, the experience of this horrific violence brought significant shame to her family to the level where she is more concerned about her children’s psychological health and dignity than her own health. Two of her adult children, who live in a different place, immediately joined the militia upon hearing what happened to their older mother. She states they were so angry and directly went to the battlefield without informing her. “I *am stressed because I cannot take care of my children*. *I know God does have a reason for everything that happens in life*. *I am not irritated*, *but I am sad because of the condition of the children*. *They have no one to take care of them*. *Their grandmother is old*, *and she also needs support*. *I left them now with her*. *But she is not able to take care of them*. *I know I should be hospitalized*, *but where will I leave my kids*, *and who will take care of them*? *Yet*, *I thank God because they did not kill my children or me*.” Rebeka was in the process of arranging medical treatment in Gondar city through the Amhara emergency fund during the interview.

#### Participant 3

Tesfa, a 10^th^ grader before the war, had been moved to the nearby village with her family when they heard about the Tigray rebel force approaching their village. However, she and her family return home after a couple of days of searching for food. She says, “*We were forced to get back home since we never took anything from home because we were shocked by their arrival*. *But when we returned*, *they used our house as a camp*. *Until they were defeated*, *they were kind to us*. *They always tell us that they will not harm civilians*, *especially farmers*. *We believed them*. *They tricked us*. “The Tigray rebel force used their house as a camp for 16 days until the Ethiopian government force made a counterattack and defeated them. Tesfa said that the TPLF forces who had settled in their house became aggressive and attacked the village on their last days. She said: “O*n Nehasie 28 (September 4)*, *they changed into a beast and attacked us*. *They harassed us*, *cursed us*. *They said that the Amhara special force had raped their children*, *sisters*, *and elders*, *and they would take revenge*. *One of the guys came to me*. *He beat me everywhere in my body and cursed me*. *He shouted at me*, *saying*, *“your people raped a monk*, *your people killed a child*, *your people raped little girls*, *your people*, *and this is a price that you should pay as revenge for your people’s action*,*” when I begged him to leave me*. *He spat on me and told me that he would cut my body into pieces holding a knife around my neck*. *He bit me with an oyster of the weapon*. *I was unable to defend myself*. *He raped me while he was biting me and cursing me*. *And he told me that he will take me with him*, *and I am his wife from now on*.”

When she was asked how she was feeling and doing at the interview, Tesfa replied:

“*The memory of the knife and the weapon always distracts me more than the rape*. *I cannot sleep at night because I have nightmares about being stabbed by a knife and struggling to escape*. *I will start running in the middle of the night*, *saying run*, *they are here; run for your life*. *Every night in my dreams*, *I will find myself hiding and running away from the TPLF*. *I always wake up crying*. *And my family will hold me and calm me down*. *I used to walk for five hours on foot to Debark every day to sell some goods and get money for my education*. *But I cannot do that anymore*. *The flashback of being spat at*, *cursed*, *strangled*, *and threatened by the knife and the weapon is a painful memory for me more than the rape itself*.”

Regarding the question about what is available to support her, Tesfa’s response shows the lack of emotional support and the challenge of dealing with the social stigma about rape. She said:

’’There *is no one that I can talk to*. *I do not have friends that will keep my secret and share my pain*. *I cannot tell people what happened to me*. *I cannot even talk to my family about what I have been through*. *I wish I were not born at all*. *I wish I had died right there when he raped me*. *My family tells me that I disgraced them*. *And the other members of the community*, *even my mate*, *call me “Leftover of the Junta*, የጁንታ ትራፊ*’’ everyone curses me and laughs at me*.”

While Tesfa deals with this horrific story, she cries for help and approaches the crisis intervention team that traveled to their village to provide her with some support. She said, *“I was supposed to hide what happened to me*. *But I sought help*, *and I came here*. *I cannot go back to the community with all their judgment*.*”*Tesfa was asked about her experience after the war. Her response shows that her life was ruined due to the sexual violence that happened to her, including losing her dream and hope for her future. She stated, “I *was engaged before the TPLFforce-controlled Chena*. *I was planning to get married after finishing my studies*. *But my engagement is canceled*. *I cannot face my family and the community*. *I cannot bear to be reminded of that day*.*”*

### Cross-case analysis

Using van Mannen’s four lived existential analytical categories (i.e., lived space, lived body, lived time, and lived others) with the associated meaning made and Bronfenbrenner’s bioecological model of risk and protective factors, we provide a cross-case analysis of themes similarities, and difference (See Table 3). Some findings emphasize the lived existential of being victims of war, rape, and sexual violence. Some conclusions emphasize risk factors in the environment (s) of the participants who experienced sexual violence and war rape. Some of the findings emphasize protective factors that appear or need to be constructed in the environment(s) of the participants to mitigate the experience of war, rape, and sexual violence and cultivate healing of their physical and psychological wounds.

**Table 3:** Themes similarities and differences of the cross-case analysis.

Categories	Themes Similarities and Differences
Lived space (spatiality)	You feel limited You feel scared and lonely Loss your safe place Moved to the unknown place
Lived body (Corporeality)	Having sleep disturbance Having nightmare Having flashback Having pain in your body (e.g. Fistula) Having increased physical reaction You feel hopeless You feel losing your dignity You feel scared
Lived time (Temporality)	Living with a new community Unable to work Losing hope to future You feel like you will never be back your old self You feel your future changed (e.g., canceled marriage plan)
Lived others (Relationality)	You feel isolated and lonely You feel disconnected from your community Feeling being a burden to family Change the family role and dynamics (e.g. grandmother being the primary caregiver Emotional disconnect from your family
Risk factors	Stigma The culture of shame Rejection Community silencing behavior Lack of community support Lack of understanding the effect of rape
Protective factors	Faith and spirituality Self-resilience Understanding and supportive family Medical and professional support Communal meaning-making process (e.g. not personalizing)

## Discussion

Using a phenomenological inquiry, the present study explored the lived experiences of war rape and sexual violence victims in Amhara Regional State, Ethiopia. The discussion was based on the guiding questions, (1) What is the lived experience of wartime rape victims in Amhara Reginal State? And the four associated subsidiary questions: What are the lived existential (lived body, lived time, lived space, and lived others) of being a victim of war rape in the Amhara Region in Ethiopia? (2) What risk factors exist in the environment (s) of girls and women victimized by war, rape, and sexual violence? And (3) what protective factors appear or need to be constructed in the environment (s) of girls and women victims of war, rape, and sexual violence?

**RQ #1: What are the lived existential (lived body, lived time, lived space, and lived others) of being a victim of war rape in the Amhara Region in Ethiopia?**

### Lived body; the physiological and psychological effect

#### Physiological effect

Some of the main themes from the data analysis reflect the victims’ lived body experience, including suffering from a mass of physical consequences. The physical symptoms reported by the victims include developing fistula, persistent ongoing pain, headache, weakness, and losing physical strength. Such physical sufferings are not uncommon given the goal of wartime rape and sexual violence as weapons of war targeted to humiliate communities by raping women and girls at gunpoint, beatings, and physical torture. Physical effects such as traumatic gynecologic fistulae are commonly reported [20]. A systematic review of 20 studies drawn from six countries, of which 18 were from five African countries, reported traumatic genital injuries/tears, pregnancy, rectal and vaginal fistulae, sexual problems/dysfunction, and sexually transmitted diseases [9]. Such physical effects are also reported to be long-standing [21].

#### Psychological effect

Even though the data from this study is limited to showing the whole psychological dimension of the victims’ experiences, it is clear that emotional symptoms that could lead to psychological problems are endorsed in their report. The research participants’ ongoing psychological pain reported included flashbacks, nightmares, memory loss, difficulty sleeping, and intrusive thoughts consistent with post-traumatic stress disorder (PTSD) symptoms. They also shared their experience of being easily triggered and feeling scared quickly, hopeless about their future, and the psychological consequence of *losing their dignity*. The family members of the victims are also at considerable risk of psychological crisis. One of the participants shared that her youngest children, who witnessed her gang rape, were crying and traumatized by the experience.

In contrast, her adult children, who heard about their mothers being raped, developed a wave of explosive anger. They went directly to the battleground and joined the military group to fight against TPLF. These psychological effects entail wartime rape and sexual violence, resulting in vast and long-ranged mental health impacts.

The present study’s findings are consistent with 14 out of 20 studies in a systematic review (Ba & Bhopal, 2017), where mental health outcomes of rape and sexual violence among civilians in conflict settings were reported. The most frequent outcomes include post-traumatic stress disorder, anxiety, and depression. Our findings are also comparable with a study conducted on adolescents in eastern Congo. Participants who considered sexual violence a non-consensual sexual experience reported more traumatic hyper-arousal and intrusion symptoms than those labeling the sexual violence as rape [22]. Furthermore, a study conducted on victims of war rape and sexual violence after 20 years of the Bosnia and Herzegovina war reported mental and general health conditions to grave concern. A substantial proportion of the victims have been suffering from clinically relevant PTSD symptoms, depression, and anxiety, with the majority, reported the rape experience ultimately affected their present life [21]. A rape survivor from the Kosovo war explains how deeply the psychological victims suffer. She says, “Rape is a wound that will burn your soul. It will make you ashamed in front of your family. You will carry it all of your life” [23].

### Lived time- the immediate effect in the now and then

Wartime rape and sexual violence differ from the actions or transgression of a few bad people. They are the byproduct of strategic, systematic, and calculated tools of war, ethnic cleansing, and genocide. It is documented that such acts intimidate women and communities, destroy specific groups’ social fabric and cohesion, and even act as a final act of humiliation before killing the victim [6]. The three women’s experiences before, during, and after wartime rape and sexual violence unfold severe psychological and social ramifications. Before the war and the horrific gang rape experience, the participants lived a functional life with a different plan for their future, including canceling marriage, spending more time seeking medical and psychological treatment, discounting their schooling, and being slow in their day-to-day functioning.

### Lived space: Effect on the action and reaction of victim space

Regarding their lived space, the participants revealed that they were forced to move from their home and community to communities unknown to them because they felt scared of people who continuously stigmatized them. This is in line with the study from northern Uganda, where among other reasons, community stigma caused wartime rape survivors to suffer from unresolved and untreated trauma, lack access to mental health care, and face economic hardships [8]. Social stigma after experiencing wartime rape is not limited to the survivors alone. It also affected their family members who witnessed the wartime rape and sexual violence, including secondary traumatic stress, memory repression, and faith. It disrupted relationships after survivors disclosed their experiences or if they had children born of wartime rape [10]. Systematic reviews where a substantial proportion of African studies included also reported social outcomes of wartime rape, such as rejection by family and/or community and spousal abandonment [11].

Our findings show that participants experienced overt stigmas (i.e., being forced to move, separated from their safe place, and moved to unknown areas) and covert stigma (i.e., feeling scared, isolated, and afraid of rejection). This is also in line with the study conducted on survivors of sexual violence in the Democratic Republic of Congo [24]. Using confirmatory factor analysis of the revised stigma scale, this study identified a couple of stigma typologies: discrimination-related stigma (i.e., enacted stigma) to which eight items (i.e., feeling of worthlessness, feeling detached from others, feeling badly treated by family, feeling badly treated by the community, feeling shame, feeling rejected by everybody, feeling stigma, and wanting to avoid others or hide) and perceived and internalized stigma to which four items (i.e., abandoned/thrown out, rejected by family, rejected by husband, and forced to leave away from your children) were loaded [24].

Similarly, another qualitative study from the Democratic Republic of Congo (DRC) also indicated the degree of rejection and abandonment by family among sexual violence survivors [25, 26]. Following experiencing social stigma and rejection, our phenomenological inquiry also explored that the participants experienced a range of new environmental challenges they had to face in their respective spheres of lived time. Accordingly, they had to start to adapt to living in new communities. Such adaptation to physical and psychological wounds is more complex than regular adjustments. Such transitions instigated even by adverse incidents such as sexual violence and rape are more stressful and impact individuals’ mental health. According to Godfrey [27], moving to a new place is among the top five most stressful situations people experience across a lifespan. We tend to develop attachments to our houses and communities that can be as strong as our relationship with our families. In the case of wartime rape and sexual abuse, survivors leave their houses, communities, and families.

### Lived others- social life and relational effect

One of the most devastating effects of war rape is its damage to the social capital on which communities are built. In her recent visit to Ethiopia, Amina Mohammed, Deputy Secretary-General of the United Nations, shared her observation at a press conference that the long-standing Ethiopian social fabric is being torn apart as manifested by sexual violence, ethnic cleansing, and hostility seen in the north Ethiopian civil war [28]. In the present study, participants’ narrative shows that living in isolation, disconnecting from their community, and not being emotionally available in their familial relationship are measured consequences of being victims of sexual violence and rape. Similarly, studies were conducted among rape survivors in conflict zones such as the DRC [29].

The gang rape occurred in a public place under the knowledge of the victim’s family and the villagers. This horrific incident is under public knowledge, magnifying its impacts on the victims’ and their families’ cultural, social, and psychological ramifications. This is similar to rape survivors of the Rwandan genocide, where the survivors’ physical and psychological injuries were reportedly aggravated by the sense of isolation and ostracization [30]. This feeling forced them to avoid revealing their experience publicly, fearing that their family and the wider community would reject them.

Like the majority ethnic groups in Ethiopia, in Amhara culture, the sanctity of a woman’s sexuality is highly valued. As one of the participants said, “*The core of my self-worth is destroyed not because of the rape*, *but because my children and others witness it*.*”*

This public occurrence of gang rape and dishonoring of the valued women in the village also destroys the entire underlying social order of the community. The suffering of war rape victims Amhara women and girls are comparable with the major themes identified by Hagen and Yohani [31] in their critical review of “The Nature and Psychological Consequences of War Rape for Individuals and Communities.” Public occurrence is one of such themes that distinctly shows how, where, and in front of whom the rape is performed as a distinguishable feature of war rape. Accordingly, it occurs in front of other women to instill fear, other soldiers to promote solidarity, and other community members to show complete suppression [31]. The situation of the present victims is also similar to different conflict settings, such as in the Republic of Congo, where a 16-year-old girl discloses, *“At some point*, *my mother and brother were brought in and forced to watch”* [31].

**Research Q #2) What risk factors exist in girls’ and women’s environment (s) victimized by war, rape, and sexual violence?**

Three themes emerge from the analyzed data related to the risk factors that exist in the environment that might hinder the recovery and healing of girls and women victimized by wartime rape and sexual violence. These are stigma, lack of legal, societal, and governmental support, and societal silencing behavior.

#### Stigma

In Amhara culture, with strong customs and taboos regarding virginity, sex, and sexuality, there is nothing highly stigmatized than rape. This study shows that their own community and villagers viewed them as dirty, unclean, and damaged. Hence, the participants suffer from disownment and isolation. Two of the three participants were forced to leave their own village because of the stigma. Thus, the women’s stories in this study unfold how rape as a weapon of war has a broader social phenomenon that furthers the women’s suffering by re-victimizing and lifting them to feel even more shame and guilt for being raped. In this connection, Adama Dieng, Active Special Representative of the Secretary-General on Sexual Violence in Conflict, once stated that *“Aggressors understand that this crime attacks individual and collective identity*, *social relationships*, *and status*.*”* And *“Victims who might survive rape often did not survive its social repercussions*.*”* He also adds, *“Many were literally dying of shame*, *forgoing medical and legal help to avoid humiliation”* [32].

Besides the exclusion, the stigma also impacts the victim’s future. For example, society views the victim as a person with damage and incapable of a future relationship. In one of the participant’s cases, we can see that the wedding plan and the marriage arrangement are also canceled because of the stigma attached to a rape victim. Other studies also showed that disclosing a sexual assault to a romantic made survivors ashamed and belittled by their partners [33]. Studies in the Kivu region of Eastern DRC also showed that many men should reject their wives after rape [34].

#### Lack of governmental or community support

Another risk factor that unfolds in this study is the victim’s lack of society or government support. The lack of legal victim protection for women against verbal, physical, and emotional abuse produces more fear among the victims and slows down their healing process. The victims report that they experienced exclusion in a social context and have to stand up to protect each other from community-based exclusion. In our study, the story of one of the participants, a mother of seven children, shows that her suffering is only magnified because she was left to support her children alone while dealing with Fistula and being ostracized in dealing with a traumatic recovery. Participants shared the lack of or limited healthcare access after the rape in the area and the stress that comes along with it. To get the medical treatment, she was forced to leave her children to her elderly mothers, who might not be able to take care of herself, let alone her grandchildren. Our finding is similar to wartime rape and sexual violence victims of various countries such as Kosovo [23], Sierra Leon [35], and Syria and South Sudan [36], where victims face a lack of government support, lack of transitional justice, lack of reintegration into their communities and marginalization.

#### Societal silencing behavior (Unspeakability)

The participants of this study also revealed that either their own family or the community didn’t come to them openly to discuss what happened to them, which led them to keep silent about it. This absence of communication and speaking openly of this horrific action made them feel disowned by their own community. According to scholars in the field, silencing the experience of sexual violence signifies psychological repression [37]. Furthermore, the inability to discursively frame the nature of this horrific experience seems to contribute to the silencing behavior. One of the participants described that even though her experience is not hidden from her family and friends, no one knows how to talk about it. She felt some indescribable fear from the silence. Similarly, a study on the impact of disclosure of rape reported that rape victim women who break the silence and speak out about their experiences at the beginning quickly consider this decision and choose to stop speaking [38]. This may be due to blame from others, being denied help, or being told to stop talking about their rape experience [36].

**Research Q #3 What protective factors appear or need to be constructed in the environment of girls and women victims of war rape and sexual violence?**

In this study, protective factors are viewed as factors that appear in the environment that help or cultivate the likelihood of survival, recovery, and healing of girls and women who are victims of war rape and sexual violence. These factors can exist at the individual, familial, community, and societal levels. Three major themes emerge from the analysis data that might answer the question about protective factors. These themes include individual and familial-resilience behavior, faith and spiritual practice, and medical and professional support.

#### Personal and familial -resilience

*The victims’ story* reflects the strength that already existed at the individual and family level. One of the participants revealed that the support she got from her mother helped her keep moving regardless of the horrific experiences. Such support also builds self-resilience in her. Similarly, their wisdom sheds light beyond the dark when mothers are resilient. Mary Barra beautifully explained this, saying, *“When I was a young girl*, *and something difficult happened*, *I would look to my mother for comfort and guidance”* [39]. The resilience of the participants of this study is indicated in their meaning-making of the event. For example, two participants believed that this horrific event was an attack on their country, ethnicity, and community more than an attack targeting their individual selves.

#### Faith and spirituality

A terrible life experience such as wartime rape increases the risk of mental health problems. Research shows that faith and spirituality may prevent the negative impact of such adverse experiences [40]. One of the three participants described the connection of faith and spirituality to the experience. She believed that she had been called to this suffering at 56 years old but was given the ability to take it day by day. All of the study participants identified themselves as Ethiopian Orthodox Tewhido Christians. They believed their faith was a way to escape the psychological and physical pain they were dealing with after the war and war rape. Our findings are comparable with research findings on these issues. For instance, faith and spirituality were found to be instrumental coping strategies in persons under challenging circumstances, such as female aid workers working in conflict zones [41], for preventing and recovering from substance abuse [42], and have an impact on mental health conditions.

#### Medical and professional support

Physical conditions such as children born out of Traumatic fistulae [20], as well as psychosocial conditions such as mental illness [21] and stigma [23], are commonly reported problems in wartime rape and sexual violence. Participants of the present study had to rely on the medical and professional community, which played a vital role in restoring healing for them. Although it was difficult for the participants to access medical facilities immediately after experiencing rape and gang rape due to the war, they could get support from health professionals after their community got free from the rebel forces. The participants extended words of tribute to organizations such as the Amhara Emergency Fund that facilitated the medical support. The psychosocial support group from the University of Gondar and Debark University also played a pivotal role.

### Idea for future research

Over the past decades, the prolonged psychological and developmental effects of war rape on victims on a global scale have been documented. The most common form of understanding is the biopsychosocial effect of wartime rape which includes trauma-related disorders such as PTSD and acute stress disorder, anxiety, depression, chronic illness, major economic disasters, and inability to be independent. While the result of this study also relates to the powerful narrative about the effect of war rape on individuals, the approach of seeing from the individual’s perceived experience only might not capture the complete picture and do justice to the victims’ experiences. Especially in a country like Ethiopia, where the culture is collective, it is essential to design research that gathers data from multiple resources across different timelines. Research shows that there is potential growth after enduring a traumatic experience. This study explores the potential protective factors that promote healing and resilience. This result indicates the need for future research on resilience and posttraumatic growth development of wartime rape survivors.

Wartime rape affects the individual, the family, and the community to which the victims belong. The result of this study reflects not only the biopsychosocial effects on the individual life and the ripple effect it has on the community at large; it breaks the existence of families. It destroys the connection and bond between families and communities. The social and community bond restoration must be included in the center of the healing process in the cultural settings. On the same line, we recommend research in the future to consider exploratory studies involving significant stakeholders such as key community members, families, health professionals, and government bodies. This study also indicated the need to explore the cultural and contextual factors (e.g., culture-specific communal rhythms, development work, counseling, and psychotherapy) for healing individual and collective trauma in resource-poor settings.

### Strengths and limitations of the study

Specific contexts such as sample size and timing need to be addressed as the strength and limitations of this study. First, this study’s result was drawn from a small sample size. It can’t be generalized to all girls and women of war rape and sexual violence victims in the Amhara region in Ethiopia. Second, the study was conducted shortly after the victims experienced the gang rape and were in the middle of facing the immediate consequence of this horrific experience. This context allows the researchers to observe the direct effects of wartime rape on the victims’ lives. The study provides a detailed and unique description of the phenomena of being the victim of war rape from girls’ and women’s perspectives. However, this context can also be a limitation since it doesn’t show the whole process of the victims’ journey to healing and meaning-making of their lived experiences across time. For example, participants were still dealing with the aftermath of this horrific event and could not use the concept of time to compare their old life to new life, with the wartime rape/gang rape as the turning point. The nature of a retrospective study is that participants are asked to recall the essential details while they might encounter suppression or forgetting.

In addition, this study doesn’t show the magnitude of the problem of rape in the war, but it can demonstrate the in-depth, severe effect that war rape has on an individual and family life. As a phenomenological research design, there might be a probability of research-induced bias influencing the studies. We used a "silencing process, “i.e., intentionally writing our bias before the interview and data analysis to mitigate the potentially damaging impact of preconceptions that may influence the research process. The interviews were conducted in Amharic, the local language, which may lead to a loss of information during transcription and translations from Amharic to English. To circumvent this problem, we got support from two language experts to help us maintain meaning and quality in the translation and back-translation processes.
